# Investigating Neuroanatomical Features in Top Athletes at the Single Subject Level

**DOI:** 10.1371/journal.pone.0129508

**Published:** 2015-06-16

**Authors:** Marco Taubert, Uwe Wenzel, Bogdan Draganski, Stefan J. Kiebel, Patrick Ragert, Jürgen Krug, Arno Villringer

**Affiliations:** 1 Max-Planck-Institute for Human Cognitive and Brain Sciences, Leipzig, Germany; 2 Institute of General Kinesiology and Athletics Training, University of Leipzig, Leipzig, Germany; 3 LREN, Département des Neurosciences Cliniques, CHUV, Université de Lausanne, Lausanne, Switzerland; 4 Department of Psychology, Neuroimaging Center, Technical University, Dresden, Germany; Max Planck Institute for Human Cognitive and Brain Sciences, GERMANY

## Abstract

In sport events like Olympic Games or World Championships competitive athletes keep pushing the boundaries of human performance. Compared to team sports, high achievements in many athletic disciplines depend solely on the individual’s performance. Contrasting previous research looking for expertise-related differences in brain anatomy at the group level, we aim to demonstrate changes in individual top athlete’s brain, which would be averaged out in a group analysis. We compared structural magnetic resonance images (MRI) of three professional track-and-field athletes to age-, gender- and education-matched control subjects. To determine brain features specific to these top athletes, we tested for significant deviations in structural grey matter density between each of the three top athletes and a carefully matched control sample. While total brain volumes were comparable between athletes and controls, we show regional grey matter differences in striatum and thalamus. The demonstrated brain anatomy patterns remained stable and were detected after 2 years with Olympic Games in between. We also found differences in the fusiform gyrus in two top long jumpers. We interpret our findings in reward-related areas as correlates of top athletes’ persistency to reach top-level skill performance over years.

## Introduction

World class performance in athletics is thought to be a consequence of individual genetic predispositions and environmental factors such as deliberate skill practice [1]. Deliberate practice is distinct from the everyday work-play situation and depends on concentration, optimized training strategies and feedback [2]. While short-term motor practice influences human brain structure and function [3], a minimum of ten years of deliberate practice has been suggested to reach internationally competitive performance levels [4]. Using neuroimaging, sports-related differences in cortical and subcortical brain morphology were observed at the group-level in female ballet dancers [5], golfers [6], karate and endurance athletes [7,8], gymnasts [9], divers [10], jugglers [11], basketball players [12] or track-and-field athletes [13]. Considering the fact that these studies looked for expertise-associated structural differences at the group level, they may fail to detect structural brain alterations linked to the individual performance level of an athlete [14,15]. So far, specific brain features were described for single patients suffering from neurological disease by comparing their individual structural brain scan (i.e. a T1-weighted MRI) to a group of healthy control subjects. Using this technique, structural abnormalities were observed in both cortical and subcortical regions in herpes encephalitis and Huntington patients [16,17]. To the best of our knowledge, specific features in the healthy individual brain of an elite athlete were not yet reported.

Skillful motor action is coordinated by subcortical and interconnected cortical brain structures such as the cortico-striatal and cortico-cerebellar pathways [18,19]. In most athletic skills like javelin throwing or long jumping, the coordinated execution of sequences of motor actions is crucial for successful task performance. Experimental evidence and theories of motor learning suggest cortico-striatal pathways to be relevant for sequential motor actions while cortico-cerebellar pathways mediated motor adaptation [20–22]. The striatum is the main input structure of the basal ganglia and possesses massive connections with cortical and thalamic centers, thus, forming specific cortico-striatal-thalamo-cortical loops [23] in which striatal and thalamic regions quickly convey neural information to motor output structures in the brainstem [24,25]. Due to their regulatory and mediating roles for efficient motor control, we expect a high computational demand on motor cortical, striatal and thalamic brain structures during motor skill-related information processing. We hypothesize that top-athletes will show structural differences in cortico-striatal circuits. For MRI data analysis we use voxel-based morphometry [26] and case-control study designs [16,27] to assess individual brain anatomy differences of three outstanding track-and-field athletes (javelin throwing and long jumping). In addition, the javelin thrower was tested twice with an interscan interval of approximately two years to assess the robustness of these effects against changes over time.

## Methods

### Subjects

#### Top-level athletes

The study was carried out in accordance with the Declaration of Helsinki and had been approved by the Ethics Committee of the University of Leipzig. Subjects gave their written informed consent prior to participation. Brain imaging and behavioral data were acquired from three track-and-field athletes (27, 28 and 30 years of age; all males). At the time of MRI scanning, these athletes performed at the highest national and intermediate international level: Two of them are long jumpers and sprinters with personal bests in long jump of >8 and >7.90 meters and each with < 11 seconds in 100 meter sprint. One of the long jumpers is a former national vice-champion. The third athlete is a javelin thrower with a personal best mark of >80 meters. The javelin thrower is a former national champion. All three athletes were active in their disciplines for at least thirteen years and conducted at least seven training session per week. Two athletes regularly participated in athletic competitions including World Championships and Olympic Games. Each of the three athletes was used for a separate case-control comparison in which deviations in brain morphology from the normal population are computed by comparing each athlete’s structural brain image (case) to an age-, sex- and education-matched control sample (control).

#### Athlete-specific control sample

To compute significant deviations from the normal population in each athlete’s brain, T1-weighted MR images from healthy control subjects were obtained from the local MRI database at the Max-Planck-Institute for Human Cognitive and Brain Sciences. Our search yielded 46 matched control subjects for the javelin thrower, 58 matched control subjects for one long jumper and 58 control subjects for the other long jumper. We ensured that all athletes and control subjects were scanned at the same MR scanner (see *MRI data acquisition*) and control subjects were carefully selected against the following criteria for each individual top athlete (see *Statistical analysis*). By controlling for the following factors, we aimed at controlling expertise-related brain features for age or educational background: age (±2 years), sex, education (A-level). We ensured that the control subjects had a level of current physical activity below 2 hours per week and received no former athletic or musical training (did not learn to play a musical instrument in the past and do not currently play a musical instrument).

#### Up-and-coming athletes

An additional group of three up-and-coming long jumpers was recruited to show that structural features in the top athletes are not present in up-and-coming athletes of the same discipline. As in the three top athletes, specific brain features in up-and-coming athletes were determined by comparing each athlete to an age- (±2 years), sex- and education-matched (A-level) control sample. The three up-and-coming athletes were used for descriptive comparison with the three top athletes. The up-and-coming athletes are active for at least nine years, conduct at least five training sessions per week and have personal bests in long jump between 6.80 and 7.20 meters.

### Procedure

Each of the six athletes completed a magnetic resonance imaging (MRI) scan and a behavioral test session on a different day. T1-weighted MRI scans were acquired to assess structural grey matter density (see *MRI data acquisition*). During the behavioral tests session, a standard drop-jump test was performed to analyze general speed and jumping performance (see *Behavioral performance test*).

### Behavioral performance test

Each of the top and up-and-coming athletes was asked to follow a standard warm-up routine consisting of approximately 800 m jogging, 5 min of stretching and 6 different jumping drills. Afterwards the athletes performed three trials of the well-established drop jump test in a standardized procedure [28]. Subjects stand on a raised platform (40 cm) as close as possible to the front edge. To start the test, subjects were instructed to shift their mass to their non-dominant leg and to slide passively off the edge of the platform until ground contact with a contact platform. Then, athletes were instructed to use their arms to support the vertical jump from the contact platform and to minimize the ground contact time, which is the time from initial ground contact until foot release. All athletes were familiar with the aim of the test (to reach a maximal jump height with a minimum ground contact time) and the overall testing procedure. Ground contact and flight times were collected and analyzed using a contact plate (Sportservice Voss, Germany) and a personal computer running in-house analysis software. The best trial out of the three trials was used to characterize performance. Motor performance (ground contact and flight time) of the top and up-and-coming athletes was compared to an existing reference group of 21 subjects that are physically active at a recreational level. Subjects from the reference group did not receive MRI scanning.

### MRI data acquisition

MR imaging (MRI) data was acquired on a 3T Magnetom Tim Trio scanner (Siemens) using a 32 channel head coil. T1-weighted images were acquired using a MPRAGE (magnetization-prepared rapid acquisition gradient echo) sequence (TR = 1.3 s; TE = 3.46 ms; flip angle = 10°, FOV = 256 x 240 mm; 176 sagittal slices; voxel size = 1 x 1 x 1.5 mm). The acquisition time was approximately 13 minutes.

### T1-weighted MRI data preprocessing

Pre-processing of T1-weighted images was performed using the VBM8 toolbox (http://dbm.neuro.uni-jena.de/vbm.html), implemented in the neuroimaging analysis software package SPM8 (Wellcome Trust Centre for Neuroimaging, University College London, London, UK; http://www.fil.ion.ucl.ac.uk/spm) running under Matlab 7.7 (Mathworks) [29]. T1-weighted images were corrected for bias-field inhomogeneities, registered using linear (12—parameter affine) and nonlinear transformations, as well as tissue-classified into grey matter, white matter, and cerebrospinal fluid within the same generative model [26]. High-dimensional diffeomorphic registration—DARTEL [30] was used for improved spatial normalization. The resulting grey matter images were scaled by the Jacobian determinants (i.e. modulation) to account for volume changes resulting from the normalization process [26]. Modulation involved correction for non-linear volume changes only [31]. These maps of grey matter density (GMD) were then smoothed with a Gaussian kernel of 8 mm (FWHM). For statistical analysis, we excluded all voxels with a GMD value below 0.2 (with a maximum value of 1) to avoid partial volume effects near the border between grey and white matter.

### Statistical analysis

#### Top-level athletes

To highlight characteristic features in brain structure of individual athletes, we compared each of the three top athletes to the matched control sample (see above). All control subjects were recruited from the local MPI database. Following our inclusion criteria (age, sex, education, physical activity, musical background), we obtained 46 matched control subjects for the javelin thrower and for one long jumper, and 58 control subjects for the second long jumper (note that there were more subjects matching the second long jumper’s age range). T1-weighted images from control subjects were acquired on the same MR scanner using a 12 or 32-channel head coil. We controlled for the different head coils and age (± 2 years of the athletes’ age) in each statistical model using a separate covariate of no interest. For analysis, we assumed that the athlete’s grey matter value at each voxel constitute the mean of a population with a variance equal to that of the control group [17,32]. This corresponds to a 2-sample *t* test in SPM with equal variances of the two groups involving one scan in the first group (the athlete) and appropriate control subjects in the second group.

#### Comparison between top-level and up-and-coming athletes

A group of three up-and-coming long jumpers was used for descriptive comparison with the three top athletes (see above). For each of the three up-and-coming athletes we used the same procedure as for the top level athletes, including a well-matched control sample, specific to the athlete, to determine features in brain morphology deviating from the control group. The three control groups consisted of 31, 59 and 59 matched control subjects because the up-and-coming athletes differed in age and hence, the number of control subjects in the MPI database matching our inclusion criteria was different (see above). Results from each of the six case-control comparisons were plotted in a scatter plot involving maximum statistical values (*t*-value) for structural differences in regions of interests (ROIs) in the striatum and thalamus.

#### Regions-of-interest

**Fig 1:**
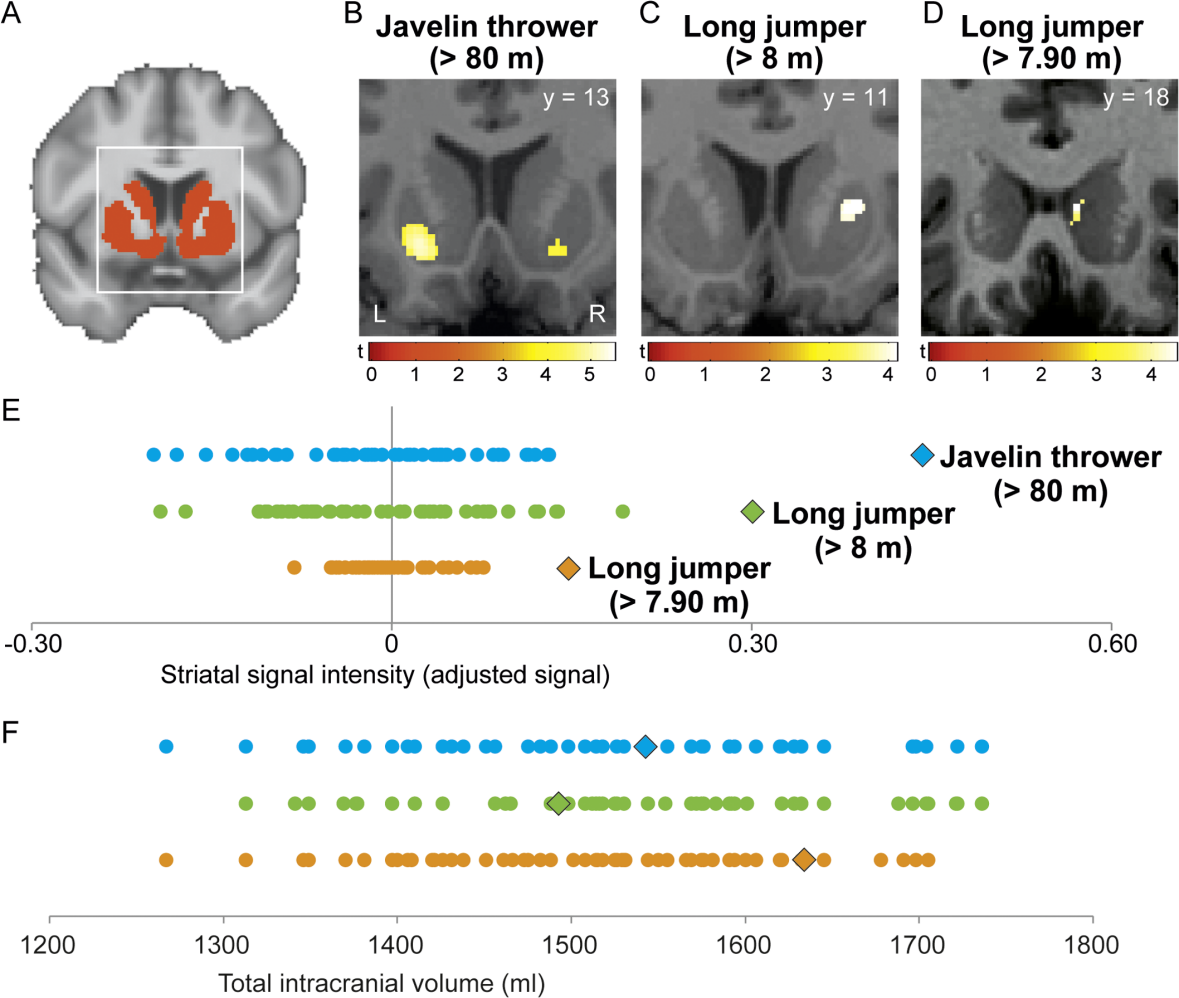
Increased regional striatal grey matter density (GMD) in top-level track-and-field athletes. (A) Template image shows striatal ROI in red. (B, C and D) Regional differences in GMD for each athlete, relative to respective control subjects, were projected onto the normalized, individual T1-weighted image of each athlete with a threshold of p < 0.05 FWE corrected. The bars show t-values. L, left, R, right. (E) Variations in striatal signal intensity for each of the three athletes (icons) and their control group (dots). Each of the three rows of dots corresponds to a case-control comparison: blue = B; green = C; orange = D. Values were derived from individual peak coordinates in striatum (B, C and D). (F) Total intracranial volume for each athlete and the control sample. Values were color- and form-coded as in E.

**Fig 2:**
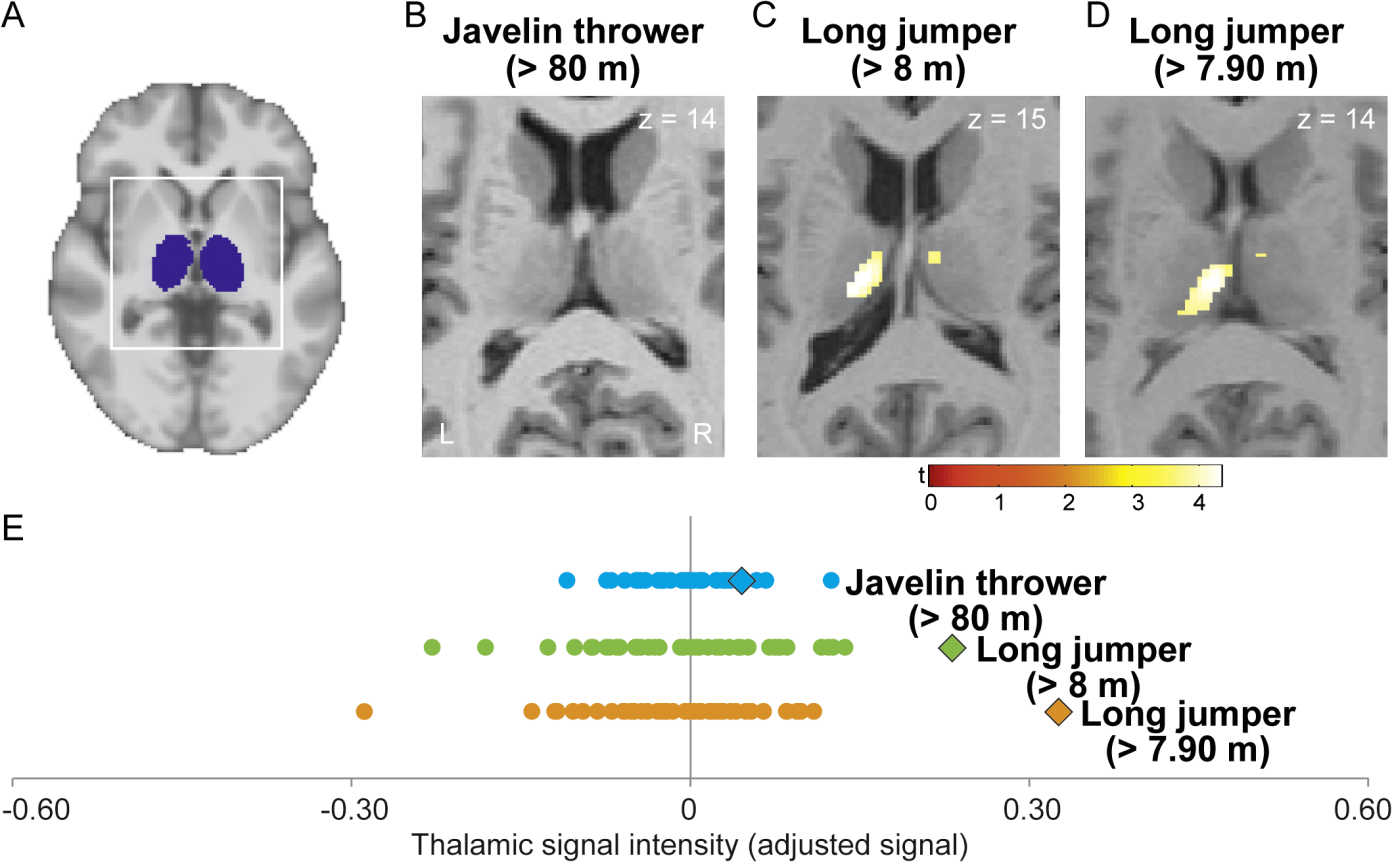
Increased regional thalamic GMD in top-level track-and-field athletes. (A) Template image shows thalamic ROI in blue. (C and D) Regional differences in GMD between each athlete and their respective control sample were projected onto the normalized, individual T1-weighted image of each athlete. No significant differences were observed in the javelin thrower (B). The bars show t-values. L, left, R, right. (E) Variations in thalamic signal intensity for each athlete (icon) and their control group (dots). Each of the three rows of dots corresponds to a case-control comparison: blue = B; green = C; orange = D. Values were derived from individual peak coordinates in the thalamus (B, C and D).

Our primary hypothesis postulates differences in subcortical brain structures of striatum and thalamus, relative to controls. Therefore, we restricted our search volume to two a-priori selected regions of interest (ROIs) in the striatum and thalamus as defined by the automated anatomical labeling atlas (WFU-Pick atlas; [33]. The striatal ROI contained the left and right caudate, and putamen (Fig 1A). The thalamic ROI was composed of the right and left thalamus (Fig 2A). Motivated by the suggestion that well-learned motor skills are stored in cortico-striatal motor circuits [18], we used two additional ROIs to test for structural differences in the sensorimotor cortex. These two ROIs covered the pre- and postcentral gyri (including primary motor and somatosensory cortices) in both hemispheres. This was done using the automated anatomical labeling atlas as described above. The first cortical ROI covered the left and right precentral gyri (PrCG) while the second one covered the left and right postcentral gyri (PoCG). Effects were reported for voxels surviving a family-wise error (FWE) corrected voxel-level threshold of *p* < 0.05. Also, trends were reported at an uncorrected voxel-level threshold of *p* < 0.001 to help formulating hypotheses in future studies. In addition, we tested for significant effects across the entire brain using a whole-brain, family-wise error corrected voxel-level threshold of *p* < 0.05.

## Results

### Behavioral test performance

The drop-jump test revealed increased flight times in both long jumpers (678 ms and 625 ms) relative to the reference group (N = 21 sport students; mean value of 452 ms ± 85 ms standard deviation). Although not tested statistically due to too few degrees of freedom, both long jumpers had also larger flight times than the three up-and-coming athletes (428, 550 and 566 ms). The javelin thrower had a flight time comparable to controls (475 ms) but a low ground contact time of 124 ms relative to the two top (134 and 145 ms) and the three up-and-coming (147, 148 and 151 ms) long jumpers (mean value of the control group of 162 ms ± 25 ms SD).

### Total brain volume

Total brain volume was comparable between each of the three top athletes (javelin thrower: 1543 ml; long jumper > 7.9 m: 1634 ml; long jumper > 8 m: 1493 ml) and their respective control sample (controls javelin thrower: 1527 ± 114 ml mean ± SD; controls long jumper > 7.9 m: 1511 ± 98 ml; controls long jumper > 8 m: 1543 ± 111 ml; see Fig 1F) as well as the three up-and-coming athletes (1489, 1756 and 1515 ml).

### Differences in regional grey matter density (GMD)

To determine unique structural properties, the brain scan of each top athlete was statistically compared to a matched control group. Separate analyses show all three top athletes had larger regional striatal GMD than their controls (p < 0.05 FWE corrected for multiple comparisons at the voxel-level; ROI for striatum; Fig 1A). In the javelin thrower, we found significant differences in bilateral ventral putamen (*t* = 5.53; x y z coordinate: -18, 9, -9; Fig 1B). This athlete was tested again after approx. two years. The same (significant) differences in bilateral ventral putamen compared to matched controls were reliably detected after this time period (*t* = 5.23; x y z coordinate: -18, 10, -9). The two long jumpers had a significant larger GMD in the right ventral putamen (*t* = 4.14; x y z coordinate: 24, 10, 4; Fig 1C) as well as in the right caudate compared to controls (*t* = 4.45; x y z coordinate: 8, 18, 4; Fig 1D). None of the three top athletes had smaller regional striatal GMD compared to controls (p > 0.05 FWE corrected for multiple comparisons at the voxel-level; ROI for striatum).

Furthermore, both long jumpers but not the javelin thrower had larger thalamic GMD relative to controls (p < 0.05 FWE corrected; ROI for thalamus; Fig 2). Significant differences between both long jumpers and their respective control samples were detected in bilateral medio-dorsal thalamus (*t* = 4.32; x y z coordinate: -10, -19, 15; Fig 2C; *t* = 4.38; x y z coordinate: -6, -18, 13; Fig 2D). None of the three top athletes had smaller regional thalamic GMD compared to the control group (p > 0.05 FWE corrected for multiple comparisons at the voxel-level; ROI for thalamus).

### Structural features in up-and-coming athletes

**Fig 3:**
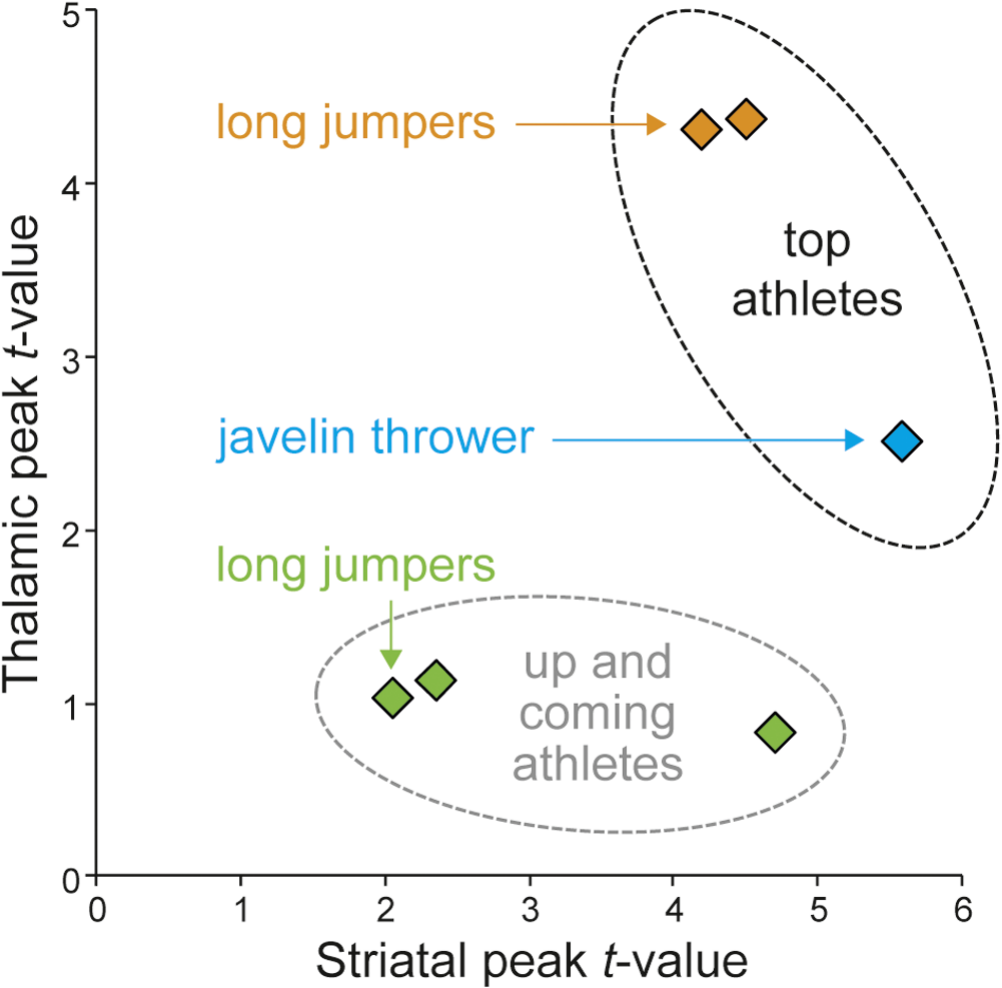
Characteristic structural features in the thalamus and striatum. The x- and y-axes show maximum *t*-values of structural differences in the striatum and thalamus for the three top athletes and the three up-and-coming athletes as compared to their controls.

Are these identified specific brain features of top-level athletes also observable in up-and-coming athletes? We analyzed brain imaging data from three up-and-coming long jumpers with personal bests between 6.80 and 7.20 meters. Similar to the previous case-control analyses, each up-and-coming athlete was compared against an age-, sex- and education-matched control group to determine structural features in the striatum and thalamus (see Methods). For thalamus, no significant differences were observed in any of the three up-and-coming athletes (p > 0.05 FWE corrected; ROI for thalamus). Structural differences in the striatum were only observed in one of the three up-and-coming long jumpers (*t* = 4.65; x y z coordinate: 10, 12, -12; p < 0.05 FWE corrected; ROI for striatum). Fig 3 summarizes the case-control results of all six athletes. The magnitude of striatal GMD differences in the up-and-coming athlete was comparable to the three top athletes. However, the two other up-and-coming athletes displayed striatal and thalamic GMD comparable to control subjects (Fig 3).

This was verified in a combined analysis of case-control comparisons from all long jumpers vs. controls including the two top long jumpers and the three up-and-coming long jumpers (5 x 2 factorial design with 5 athletes and 5 matched control groups). Within regions where we found significant effects in the separate single-case analyses (analysis of striatum, thalamus and whole–brain analysis mentioned below), the combined analysis revealed more pronounced structural differences in top as compared to up-and-coming athletes in the thalamus (p = 0.015 FWE corrected at voxel-level in 10 mm ROI around peak voxel from separate analyses, *t* = 3.42, x y z -9–16 15) and fusiform gyrus (p < 0.001 FWE corr., *t* = 4.63, x y z -32–70–8) but not in the striatum (p > 0.05). This is consistent with results from separate single-case analyses where striatal effects in the two top long jumpers were spatially more dispersed (in caudate and putamen, see Fig 1). Although, we compared athletes indirectly via case-control contrasts, this additional analysis revealed common (in both top long jumpers) and specific (in top but not in up-and-coming long jumpers) aspects of brain structural differences between individuals.

### Sensorimotor-cortical ROIs

In addition to the subcortical ROIs, we analyzed structural differences in target areas of the motor cortical-striato-thalamo-cortical pathways. We defined two regions-of-interest for the pre- and postcentral gyrus including primary motor and somatosensory (M1 and S1) brain regions that have been found to be vulnerable to training-dependent structural brain plasticity and subject to morphological differences in musicians compared to controls [34,35].

In the top javelin thrower, we found no significant difference (increase or decrease) to the control group in the precentral gryus (PrCG) and a non-significant trend towards larger GMD in the left postcentral gyrus (PoCG; p < 0.001 uncorrected at the voxel-level; ROI for PrCG and PoCG). Similarly, no significant difference for PrCG and a trend towards larger GMD in right PoCG was observed in one of the two top long jumpers (p < 0.001 uncorrected at the voxel-level; ROI for PrCG and PoCG). The other top long jumper displayed significantly larger right and left PoCG GMD (p < 0.05 FWE corrected at the voxel-level; ROI for PoCG) and a non-significant trend in left PrCG (p < 0.001 uncorrected at the voxel-level; ROI for PrCG).

### Whole-brain analyses

**Fig 4:**
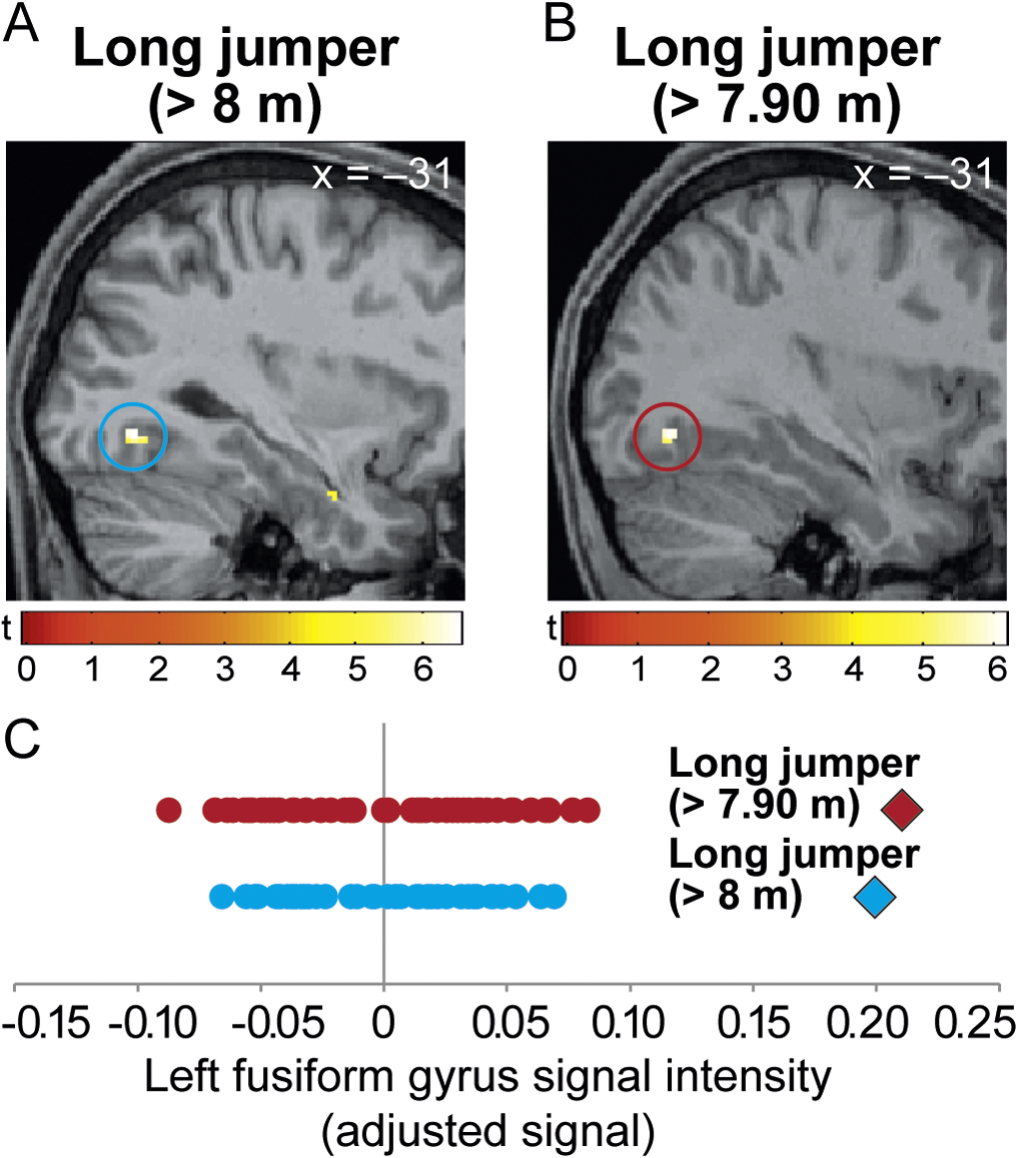
Whole-brain analysis. **Significantly larger regional GMD in the left fusiform gyrus (blue and brown circles) in each of the two top long jumpers as compared to controls (for A: *t* = 6.58; x y z coordinate: -32, -70, -8 and for B: *t* = 6.15; x y z coordinate: -32, -72, -8)**. The bars show t-values. GMD differences were not observed in the top javelin thrower or the three up-and-coming long jumpers. (C) Variations in fusiform gyrus signal intensity for each athlete (icon) and their control group (dots). Each of the two rows of dots corresponds to a case-control comparison: blue = A; brown = B. Values were derived from individual peak coordinates in the fusiform gyrus (A and B).

We also performed whole brain analyses to detect expertise-related brain features that were not part of our primary hypotheses. Surprisingly, we observed significantly larger cortical GMD in both top long jumpers but neither in the top javelin thrower nor in the three up-and-coming long jumpers in the left posterior fusiform gyrus (*t* = 6.38; x y z coordinate: -32, -70, -9 and *t* = 5.84; x y z coordinate: -32, -70, -8; p < 0.003 and p < 0.016 FWE corrected at the voxel-level across the whole-brain; Fig 4). None of the three top athletes had significantly smaller regional cortical GMD compared to the control group across the whole brain (p > 0.05 FWE corrected for multiple comparisons at the voxel-level; whole-brain analyses).

## Discussion

We tested whether individual variations in brain anatomy can be detected with single-subject MR image analyses in elite athletes, relative to a normal control group. Three top level track-and-field athletes with more than 10 years of experience in competitive sports were used as a model of physiological brain adaptation through processes of motor skill learning and optimization. Each athlete had significantly larger regional striatal grey matter density (GMD) compared to age-, sex- and education-matched control subjects. While a javelin thrower showed restricted enlargements in bilateral striatal GMD that were reliably detected before and after 2 years with international tournaments in between, two long jumpers both had enlarged striatal, thalamic and visual cortical GMD compared to controls. We did not find significant differences in sensorimotor cortical regions in any of the three athletes. Our findings show significant deviations of brain structure in single healthy individuals and future studies using a similar design need to confirm these findings.

Our findings are consistent with the view that individual variations in brain anatomy reflect differences in behavioral performance [14,36]. Correlations between motor performance and GMD have been previously found in standard laboratory motor tasks (for review see [14]). Here we tested for significant deviations in brain structure in healthy subjects capable of performing exceptional motor skills compared to healthy controls without this skill level. We are aware of the potential risk that between-subject variability, irrespective of expertise status, may confound single-case versus group comparisons. However, we believe that we minimize this risk by using a control group that is carefully matched not only for age and gender but also for educational background. The rate of false positives in single-case designs is generally higher in cortical as compared to subcortical regions [37]. By using a large and athlete-specific control sample, we minimized the risk of false positives [37] and observed significant deviations in subcortical brain anatomy (striatum and thalamus) as well as in the visual cortex that were consistently found in two long jumpers. In addition, striatal GMD differences in the top javelin thrower were reliably detected before and after a 2 year time period with international tournaments (World Championships and Olympic Games) in between. We believe that our findings may reflect GMD variations related to the expertise level and discipline of individual elite athletes (Fig 3).

In both top long jumpers, we identified considerable differences in regional GMD in cortical regions of the left fusiform gyrus (Fig 4). The execution of highly complex motor skills like a long jump may require information processing in a network of cortical regions responsible for the perceptual, cognitive and motor aspects of skill performance [38].

The long jump is a complex athletic skill that is separated into the approach phase, take-off, flight phase and landing. Long jump performance is heavily dependent on the quality of the approach phase [39]. The goal of the approach phase is to strike the take-off board as accurately as possible with an optimal running velocity and a minimum loss of speed. Striking the take-off board with both accuracy and speed is a major challenge for the performer and hence subject to intensive training [40–42]. Therefore, it seems plausible that the optimal transition from approach to flight phase depends not only on extraordinary motor but also visual abilities [40] because athletes need to accurately perceive the dynamic take-off board position to compute the body-board relationship during high-speed running. Minimal deviations in visual processing will lead to failure (trespass) or decreased performance [40]. The latero-occipital complex is implicated in dynamic object processing and responsible for the perception of shape differences during object motion [43]. Accurate object shape perception is required for take-off board recognition and subsequent foot placement during long jumps. Our results show that successful long jumpers have increased GMD in the left fusiform gyrus (Fig 4) which was specific for the two elite long jumpers and not observed in the top javelin thrower as well as in the up-and-coming long jumpers. We hypothesize that training of high-precision, visual online-monitoring of the board position during the approach phase with respect to the body and individual stride pattern is associated with higher activity and a pronounced structural composition of the left fusiform gyrus. Due to the exploratory nature of this finding, more information is needed to confirm and better understand the functional role of this brain area for successful athletic performance in long jumping.

Cortico-striatal-thalamo-cortical loops are fundamental for information processing. If we accept that performance of highly complex motor tasks involves information processing within and between widespread cortical and subcortical networks across the brain [38], reorganization of subcortical input structures that receive, transmit or relay these massive cortical signals, such as the striatum or thalamus, seems plausible and expertise-related brain differences have been demonstrated previously in group studies [44,45]. We found increased striatal GMD in each of three top athletes (Fig 1). The peak coordinates for all three athletes were located in the ventral putamen and caudate suggesting a relationship to the motivational aspects of exceptional motor performance and their development over years of deliberate practice [4,46]. Cortico-striatal loops are implicated in reward processing (motivation) and decision-making for optimal behavior [47–49]. Goal-directed behavior is the consequence of reinforcement learning where actions causing high rewards are maintained and actions with low or no reward are inhibited [48,50,51]. The career of a top athlete is directed towards success in national and international tournaments and corresponding internal thoughts or external stimuli may elicit a strong motivational response to maintain goal-directed behavior during strenuous exercise sessions or in situations of everyday life when training has priority compared to other activities of daily living e.g. social interactions outside training. Athletes must possess the so-called “rage to master” [4,46]. An appropriate performance motivation is one of the most important prerequisites to become an expert performer [46] and therefore, variations in striatal GMD could be a consequence of an altered performance motivation.

The thalamus is implicated in the control of arousal and modulation of cortical brain activity via thalamo-cortical connections [24]. During competition, an athlete has to focus attention to the trial at hand and suppress distracting stimuli coming from external sources such as the audience or competitors as well as from internal sources such as the fear of failure [2]. These influences can significantly limit the performance of athletes, especially during important competitions. Thus, athletes need to put all mental and motor effort in a limited amount of trials (in long jump and javelin: 3 trials qualifying + 3 trials final). This selective suppression of distracting and facilitation of relevant stimuli might be supported by integrity of thalamic structures and therefore subject to expertise-related structural plasticity [52].

We did not find significant differences between each athlete and controls in areas of the sensorimotor cortex, a region that is strongly associated with motor control and learning and has been found to be enlarged in group studies of musicians [35,45]. Although basic motor control demands may differ between musicians and track-and-field athletes [53,54], athletic skills like javelin throwing are characterized by the control and coordination of multiple sequences of limb movements that inherently rely on cortical motor structures [38].

Therefore, it seems that the imperative for structural plasticity in motor cortical areas is modulated by motor training regimen such as musical or athletic training. Optimization of highly automatized, athletic skills may not anymore trigger expansion of neural structures while permanent acquisition of new musical pieces over the course of a musicians career causes ongoing structural plasticity in M1 [55]. These differences at the neural level may be associated with differences in underlying learning mechanisms between musicians and athletes. Here, model-based and model-free learning mechanisms can be distinguished [56] and complex (sports/musical) skill training is mediated by both mechanisms with more impact of one or the other depending on the actual skill (athletic/musical). For example, improvements in athletic skill requires model-free learning mechanisms to consolidate motor commands that led to success in training (reinforcement learning) while model-based mechanisms dominate learning of musical pieces in which improvements in motor performance occur through updates of internal forward models based on sensory prediction errors [56]. In addition to strength, speed, endurance and flexibility, refinement of coordinative aspects of a motor skill (motor skill learning) is only one component of athletic training.

Therefore, we believe that the cortical motor system still has a critical role for complex skill performance in our athletes but the relevant neurobiological adaptations may be consolidated in a state that is no longer observable with our MRI methods. Long-term motor training may have caused a renormalization of initial M1 adaptations that were no longer visible after motor skill acquisition [57,58]. Also, the equal variance assumption in our statistical analysis [32] may have hampered our ability to find subtle differences in brain morphology between athletes and controls that nevertheless may include behaviorally-relevant changes in neural network function of M1 [59]. Extended practice leading to highly skilled performance may therefore results in a more efficient generation of neuronal activity in M1 [57].

### Limitations

Here we compared structural MRI scans of single athletes with a group of matched control subjects to detect significant deviations from average human brain structure. These anatomical differences may be associated with their profession but single case comparisons do not permit to make inferences about the cause of these differences or potential relationships to sports behavior because differences may be mediated by factors independent of an athletes profession. We made an attempt to control for confounding effects of age, gender and education by careful selection of control subjects and multiple testing of different top athletes to show similarities in structural features. Also, differences in thalamic and visual cortical structure were prominent in the two top long jumpers but not in three up-and-coming long jumpers suggesting achievement specificity of our findings. However, we cannot exclude the possibility that other potential factors have mediated our structural differences and therefore future studies are required to confirm our findings and to shed more light into the causal relationship between brain structure and athletic training in track-and-field athletes.

### Conclusion

The behavioral underpinnings of expert performance have been subject to numerous studies in sports and cognitive science in the past decades [2]. Here we demonstrate the possibility to detect neuroanatomical features in the brain of a top-level athlete using a case-control study design. Neuroanatomical features were found in three elite athletes in striatal and thalamic regions and areas of the visual but, surprisingly, not in the sensorimotor cortex. In future studies, cross-sectional designs comparing groups of athletes from different disciplines and performance levels will be important to study expertise- and discipline-specific structural brain correlates. We conclude that specific anatomical features can be observed at the single-subject level in the brain of a top athlete.
